# Is the Person-Situation Debate Important for Agent-Based Modeling and Vice-Versa?

**DOI:** 10.1371/journal.pone.0112203

**Published:** 2014-11-04

**Authors:** Katarzyna Sznajd-Weron, Janusz Szwabiński, Rafał Weron

**Affiliations:** 1 Institute of Physics, Wrocław University of Technology, Wrocław, Poland; 2 Institute of Theoretical Physics, University of Wrocław, Wrocław, Poland; 3 Institute of Organization and Management, Wrocław University of Technology, Wrocław, Poland; University of Bristol, United Kingdom

## Abstract

**Background:**

Agent-based models (ABM) are believed to be a very powerful tool in the social sciences, sometimes even treated as a substitute for social experiments. When building an ABM we have to define the agents and the rules governing the artificial society. Given the complexity and our limited understanding of the human nature, we face the problem of assuming that either personal traits, the situation or both have impact on the social behavior of agents. However, as the long-standing person-situation debate in psychology shows, there is no consensus as to the underlying psychological mechanism and the important question that arises is whether the modeling assumptions we make will have a substantial influence on the simulated behavior of the system as a whole or not.

**Methodology/Principal Findings:**

Studying two variants of the same agent-based model of opinion formation, we show that the decision to choose either personal traits or the situation as the primary factor driving social interactions is of critical importance. Using Monte Carlo simulations (for Barabasi-Albert networks) and analytic calculations (for a complete graph) we provide evidence that assuming a person-specific response to social influence at the microscopic level generally leads to a completely different and less realistic aggregate or macroscopic behavior than an assumption of a situation-specific response; a result that has been reported by social psychologists for a range of experimental setups, but has been downplayed or ignored in the opinion dynamics literature.

**Significance:**

This sensitivity to modeling assumptions has far reaching consequences also beyond opinion dynamics, since agent-based models are becoming a popular tool among economists and policy makers and are often used as substitutes of real social experiments.

## Introduction

Agent-based models (ABM) are believed to be a very powerful tool in many disciplines [1]–[8]. Traditionally this kind of approach, that takes into account how individuals interact, was the domain of statistical physics [9]. Physicists were able to describe complex collective phenomena (e.g. phase transitions) as a result of microscopic interactions [10], [11]. The models used by statistical physicists were rather simple, usually not because the physical reality was simple but because simple models were much easier to deal with and able to describe universal features. Moreover, within simple models it is possible to assess what microscopic factors are the most important from the macroscopic point of view. Agent-based models that are nowadays used in other disciplines are often more complicated, although the seminal model proposed by Thomas Schelling [12] to describe spatial segregation in societies was as simple as the simplest models in statistical physics can get. It is not the aim of this article to discuss if agent-based models have to be simple or not. It is obvious that a model should be designed to describe the problem at hand and this will determine the level of complexity [2]. However, the fundamental question that arises in all applications is how to introduce interactions.

Even if we consider only the simplest models of opinion formation – in which opinions are represented by binary variables and in which social influence is limited to conformity – we can find in the literature a number of competing and commonly used approaches, including the voter model [9], [13], the majority rule [14], [15], the Sznajd model [16] and Glauber-type opinion dynamics [17]. There have been also a few attempts at unifying these models. The first one was proposed by Galam and is presently known as the general sequential probabilistic model [18]. In 2009 the *q*-voter model was introduced [19] and it includes the voter and Sznajd models as special cases. In 2013 yet another generalization of the *q*-voter model was proposed [20]. Interestingly, the *q*-voter model belongs to the broad class of so-called threshold models [21], which have gained considerable popularity in the social sciences.

Even greater confusion prevails if different types of social influence are considered, such as conformity, nonconformity, anticonformity, etc. An extended discussion of these issues can be found in [20]. Let us only briefly mention that the presence of zealots [22], inflexibles [23], experts [5] or independent agents [20] significantly changes the output of the model and introduces phase transitions [24]. Moreover, in some papers a nonconformist behavior is introduced as an individual trait [22], [23], whereas in other as a situational factor [20], [24]–[27]. This raises the question of the role of ABMs. Certainly some of them are just interesting in themselves and can be investigated from the point of view of basic research, in the domain of non-equilibrium statistical physics [9]. However, the ultimate objective of opinion dynamics ABMs is (or at least should be) making them applicable in the social sciences. But if so, the models should be based on realistic assumptions.

These considerations nicely lead us to two questions which have been the motivation for this paper:

1. *Micro level*: What determines human behavior – personal traits or rather the situation?
2. *Macro level*: Do the modeling assumptions we make regarding social interactions (personal traits vs. situation) have substantial impact on the simulated behavior of the system as a whole or not?

Obviously answering the first question lies beyond the domain of physics. In fact, among psychologists there has been a longstanding and vigorous discussion on this topic, known as the *person-situation debate*. The debate started in the late 1960s and recently has been announced to be over (for a review see the special issue of the Journal of Research in Personality [28]). On the other hand, there is still a lot of controversy around the subject. For instance, some psychologists argue that the debate is an academic problem because the concept of situation is not well defined or the questions in the debate are poorly posed. Nevertheless, intuitively the subject of the debate is quite clear and relatively easy to describe with agent-based modeling. It seems that nowadays most psychologists agree that both, the situation and personal traits, influence human behavior and such an approach is also visible in some ABMs [29]. The problems that are still under consideration in the literature are rather related with the question what factors and when are more important [30]–[32]. It is not the aim of this paper to solve one the most significant debates in the history of psychology. For us, the debate is an excellent excuse for a closer look at some fundamental problems in the area of agent-based modeling. On the other hand, the results obtained within agent-based modeling may shed some light on the debate itself.

Let us now focus on the second question. Certainly the debate is very important for psychologists but it is also important from the macroscopic or societal point of view? Imagine that we have a group of 1000 people and consider two approaches. In the first approach 100 individuals are independent, i.e. act independently of the social influence, and the remaining 900 are conformists, i.e. follow the behavior of other group members. In the second approach each member of a group acts independently with probability 0.1 and conforms with probability 0.9. The expected value of the independent behavior in both approaches is exactly the same. A first guess would most likely be that no differences between the two approaches will be visible on the macroscopic scale. And this indeed is the reply one of us (KSW) was giving when asked on different occasions. Only recently have we realized that this problem – while very difficult, if possible at all, to solve via social experiments – can be easily addressed within a microscopic agent-based model.

## Methods

**Figure 1:**
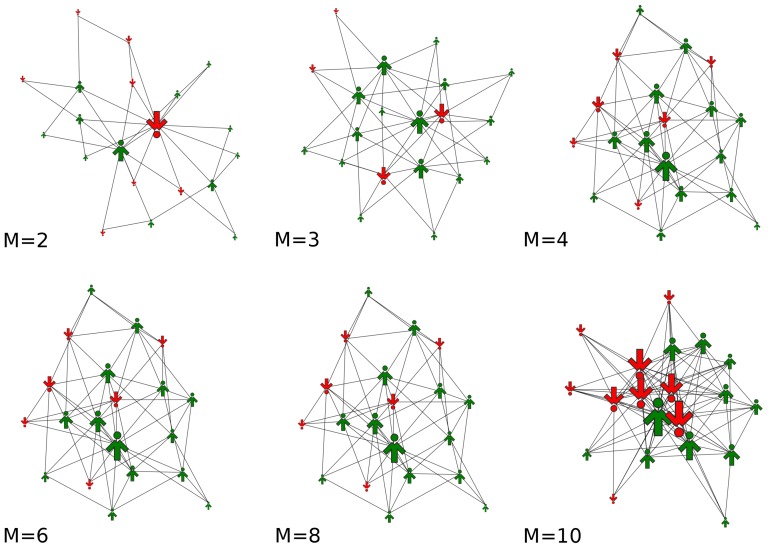
Sample Barabasi-Albert network structures for different densities of links, represented by parameter M. The agents are described by a single binary variable and called *spinsons* to reflect the dyadic nature of the agent (*spin*) and the object of study (*person*). The spinson size is proportional to the number of outgoing links, the color (and simultaneously orientation) represent the binary opinion.

To investigate the above issue we use *q*-voter models, which have been originally proposed as situation-oriented [20] but can be easily reformulated to become personality-oriented. We set 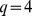 to reflect the empirically observed fact that a group of four individuals sharing the same opinion has a high chance to 'convince' the fifth, even if no rational arguments are available [33], [34]. The agents in our models are described by a single binary variable, which may correspond to 'yes' or 'no' in the field of opinion dynamics or 'adopted' and 'not adopted' when modeling innovation diffusion. For such a simple agent, Nyczka and Sznajd-Weron [20] have recently introduced the name *spinson*, which reflects the dyadic nature of the agent (*spin*) and the object of study (*person*), see Fig. 1. We use this name throughout the paper as it nicely allows to go around gender issues. It should be also emphasized that models like the one discussed here are particularly useful in the field of diffusion of innovation [6], [8], [35]. Hence, in the remainder of the paper we use the 'innovation diffusion' language.

Interactions between spinsons are very simple, although based on empirical evidence (as reported by social psychologists [34]). Like in [36], in each time step a group of four connected spinsons is chosen and if the group unanimously shares an opinion it will influence one neighbor, which can behave like a conformist (i.e. take the opinion of the group) or act independently. In the case of independent behavior, with probability *f* the spinson changes its opinion and with 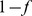 stays with the current opinion. Parameter *f* represents *flexibility*; to calibrate the model to reality it may be set equal to the level of conservatism in the society [37], [38]. Such a simple model can be easily formulated as situation-oriented or personality-oriented. In the first case – let us call it *situation* – an agent acts independently with probability *p* and follows the group with probability 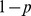. As a result, each spinson can sometimes behave independently and sometimes conform with the group. In the second approach or model – dubbed *person* – a fraction *p* of spinsons in the society are independent. The expected value of independent behavior is exactly the same in both approaches and therefore the first guess could be that both models give the same result on the macroscopic level.

## Results

### Monte Carlo simulations

**Figure 2:**
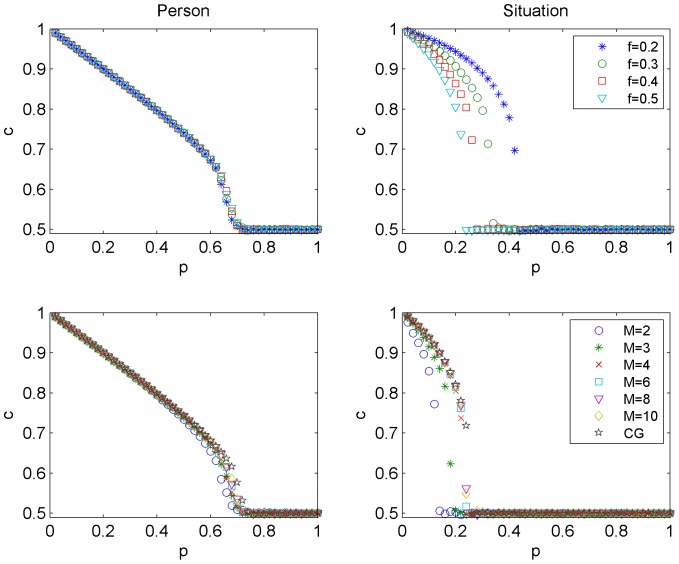
Concentration of adopted *c* in the stationary state as a function of independence *p* for the *person* (left column) and the *situation* (right column) models. Simulation results are averaged over 1000 Monte Carlo runs and concern Barabasi-Albert networks of size 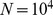. In the top row the dependence on flexibility *f* is shown for 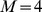, in the bottom row the dependence on *M* is shown for 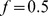. Note that the results for larger values of *M* approach the results for the CG, see Fig. 3.

We investigate both modeling approaches on Barabasi-Albert networks, as they nicely recover most of the features of a real social network [39]. We build the test network starting from a fully connected graph of *M* nodes and then preferentially attach *M* new nodes at each time step until the network achieves the assumed number of nodes *N*. We then conduct Monte Carlo simulations. In the initial state all spinsons are 'down', which corresponds to the situation prior to introducing the innovation (e.g. a tablet, a new electricity tariff) when none of the agents is 'adopted'. Due to independence some spinsons start to flip and then social influence from a unanimous group of 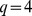 spins may influence a neighboring (and connected) spinson. Eventually the system reaches a stationary state in which concentration of adopted fluctuates around some average value 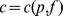. As a result of competition between social influence (an ordering force) and independence (which introduces noise and disorders the system), a phase transition appears in both models (see Fig. 2). For level of independence 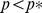 there is a state in which a majority (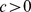) coexists with a minority and for 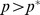 a status-quo situation is observed (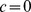). Surprisingly, in the *person* model there is no dependence on parameter *f*, which describes how often independent spinsons change their opinion. On the other hand, *f* influences the results significantly in the *situation* model (see the top right panel in Fig. 2).

Not only flexibility is an irrelevant parameter in the *person* model. Also the density of the network, represented by parameter *M*, does not influence the results (see the bottom left panel in Fig. 2). In the *situation* model the structure of the network is more important, although with increasing *M* concentration *c(p)* approaches a limiting case which coincides with the result obtained for a complete graph. One could argue that the differences between the two considered models are visible because of the differences in network structures. However, to our surprise, the results presented in the bottom panels in Fig. 2 suggest that the differences appear even on a complete graph. Indeed, as can be seen in the bottom panels, flexibility *f* is a redundant parameter in the *person* model (left panel), but not in the *situation* model (right panel).

### Analytical calculations for a complete graph

In this section we will perform analytical calculations to answer the intriguing question why the results for the two studied models differ so much even on a complete graph. In a general complex network setup, it is not easy to compute how the number of adopted spinsons changes in time and what is the stationary state. However, in the case of a complete graph this task is exceptionally simple and corresponds to the method known in statistical physics as the *mean field approach* (MFA; see e.g. [9]). On a complete graph each spinson is connected with every other spinson and therefore they are all neighbors. Hence, the system is completely homogeneous in the sense that the local concentration of adopted spinsons is statistically equal to the global concentration 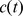. Therefore we can write down the equation that describes the evolution of the system.

Recall that in the *person* approach there are two groups of agents and that the opinion dynamics in each of these groups is different. Let 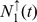 and 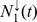 denote the number of adopted (↑) and unadopted (↓) independent spinsons at time *t*, respectively. Then the total number of independent spinsons is constant in time: 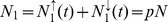. Further, let 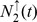 and 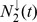 denote the number of adopted (↑) and unadopted (↓) conformists at time *t*, respectively. Similarly, the total number of conformists is constant in time: 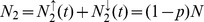. Finally, denote by 
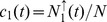
 the concentration of adopted independent spinsons at time *t* and by 
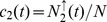
 the concentration of adopted conformists at time *t*; both are computed with respect to the whole system size *N*. Note that the ratio of adopted spinsons 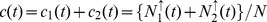.

In each elementary time step the number of adopted independent spinsons 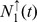 can increase by 1 only if: (i) an independent spinson is drawn from the set of all spinsons (the probability of this event is equal to 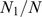), (ii) this spinson is unadopted (with probability 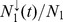), and (iii) the spinson flips (with probability *f*). Analogously, in each elementary time step 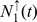 can decrease by 1 only if: (i) an independent spinson is drawn from the set of all spinsons, (ii) this spinson is adopted (with probability 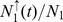), and (iii) the spinson flips. Therefore, we can write the following evolution equation for the number of adopted independent spinsons:
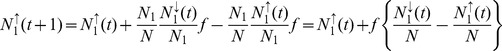


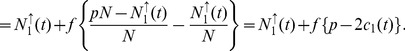
(1)


Dividing both sides by *N* we obtain the evolution equation for the concentration of adopted independent spinsons:
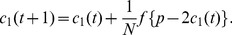
(2)


A similar reasoning can be conducted for the number of adopted conformists. In each elementary time step the number of adopted conformists 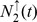 can increase by 1 only if: (i) a conformist is drawn from the set of all spinsons (with probability 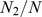), (ii) this spinson is unadopted (with probability 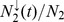), and (iii) all four chosen neighbors are adopted (the probability of this event is approximately equal to 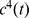). Let us briefly comment on the later statement. The exact value of the probability in (iii) is equal to:
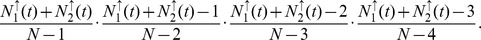



However, assuming that for 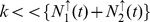 we can approximate 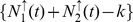 by 
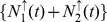
 and for 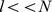 we can approximate 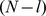 by 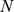, the probability in (iii) can be approximated by
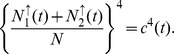



Analogously, in each elementary time step 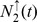 can decrease by 1 only if: (i) a conformist is drawn from the set of all spinsons, (ii) this spinson is adopted (with probability 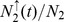), and (iii) all four chosen neighbors are unadopted (the probability of this event is approximately equal to 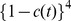). Therefore, we can write the following evolution equation for the number of adopted conformists:
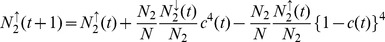


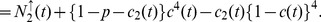
(3)


Dividing both sides by 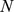 we obtain the evolution equation for the concentration of adopted conformists:
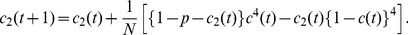
(4)


Finally, combining formulas (2) and (4) we obtain the complete set of equations which describe the time evolution of the system in the *person* model:
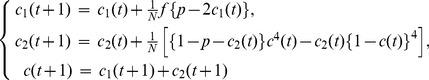
(5)


Now, let us briefly comment on the *situation* model. Within this approach all agents are homogeneous and behave independently with probability *p* and conform with probability 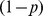. Following a similar argumentation as in the *person* model, we arrive at the evolution equation for the concentration of adopted spinsons in the *situation* model:
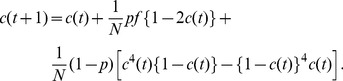
(6)


**Figure 3:**
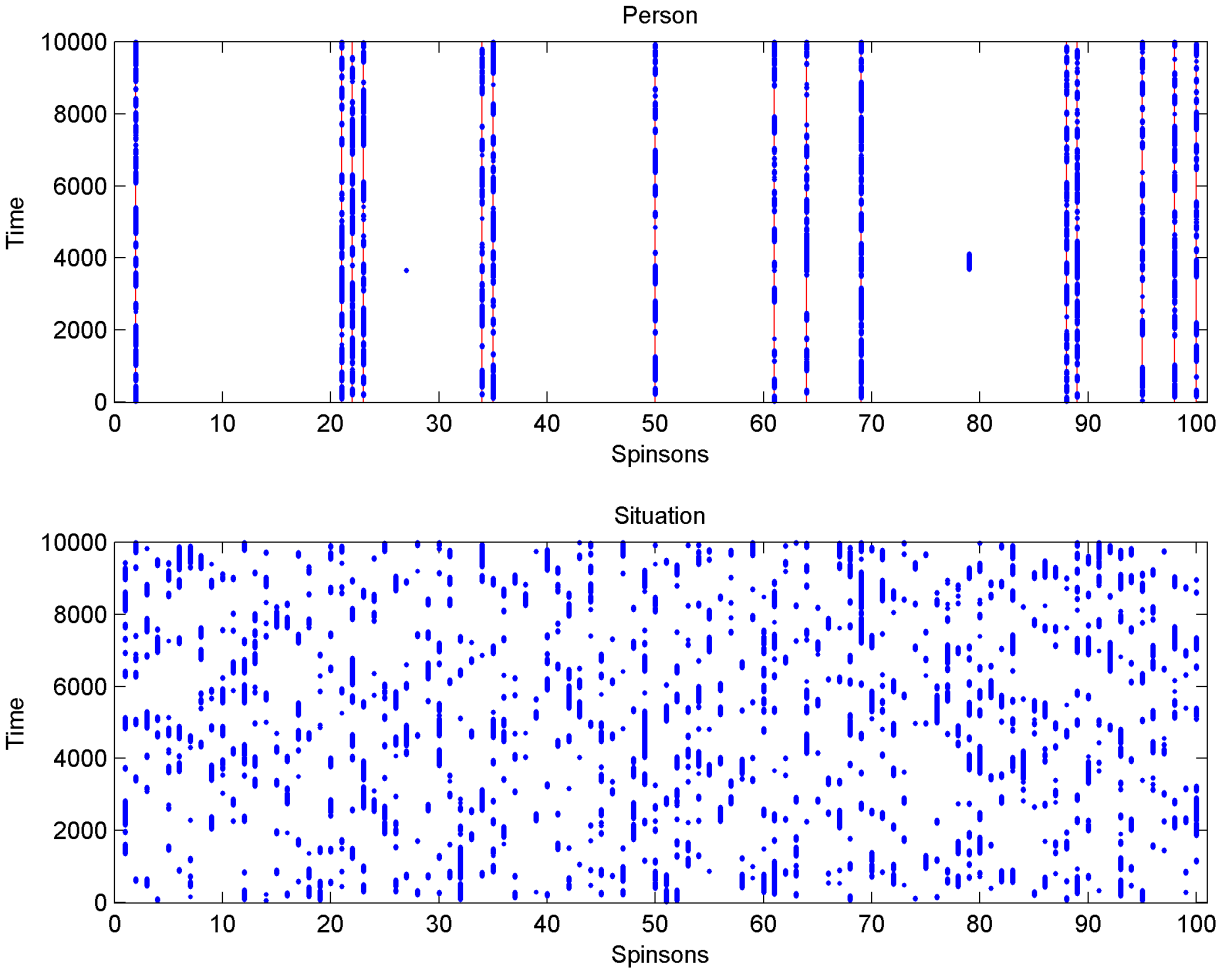
Concentration of adopted *c* in the stationary state as a function of independence *p* for the *person* (left) and the *situation* (right) models on a complete graph (CG). Analytic results obtained by iterating formulas (5) and (6) for four values of flexibility *f* are denoted by lines. For comparison, MC results for 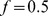 (the same as in Fig. 2) are shown as stars. Except for the neighborhood of the critical point, the stars lie on the dotted purple line. This slight discrepancy is caused by the fact that near the critical point very long simulation times are needed to reach the steady state.

From formulas (5) and (6) we can obtain the stationary value of the concentration of adopted spinsons, i.e. 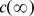, and it agrees very well with Monte Carlo results (compare Figs. 2 and 3).

But how can we understand the difference more intuitively, without looking at these two figures and the formulas behind them? To do this let us consider again a system in which initially there are no adopted. In the *person* model only independent spinsons can flip. With increasing *f* they flip more often but this is generally true only for independent spinsons (see the upper panel in Fig. 4). Only if all spinsons in a selected group of *q* agents are adopted a non-independent neighboring spinson (note that on a complete graph all spinsons are neighbors) may be flipped, which happens quite rarely for smaller values of independence *p*. On the other hand, in the *situation* model, every spinson can flip with probability *pf* and therefore with increasing *f* more and more spinsons flip (see the lower panel in Fig. 4). Therefore the results in this case depend on *f*.

**Figure:**
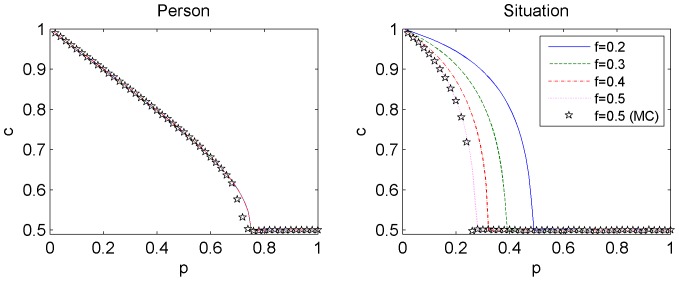
The time evolution of a system of 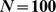 spinsons on a complete graph from an initial state of no adopted. The number of time steps is 10,000, which corresponds to 100 Monte Carlo Steps (MCS). Blue dots denote 'adopted', white spaces denote 'not adopted'. In the *person* model (top panel) the red solid lines denote positions of independent spinsons. Note how the blue dots stay on the red lines – generally only the independent spinsons flip. This is very much unlike the dynamics of the *situation* model (bottom panel).

## Conclusions

As suggested by numerous social experiments, the situation can almost completely prevail over personality [34]. On the other hand, personality psychologists argue that *there is considerable agreement that personality attributes exist and that these attributes shape how individuals adapt to the challenges of life* [28]. Although the relative importance of personality versus situational factors is very important from the point of view of psychology, it has been ignored or forgotten in agent-based modeling (at least in the context of binary opinion dynamics models). One of the reasons for such a situation may be the belief that such a detail does not affect the macroscopic behavior of the system.

However, as we have shown, there are significant differences between the personality- and situation-oriented modeling approaches. In the former case, the results on the macroscopic scale do not depend on flexibility (representing the level of conservatism in the society) nor on the network structure, which does not seem to be very realistic. This has far reaching consequences for agent-based modeling in general [7]. Some psychologists argue that the person-situation debate is an academic problem because the concept of situation is not well defined. However, within agent-based modeling this problem can be clearly defined.

Our results indicate that the situation approach may be more relevant when modeling social interactions, which – in a way – validates experimental results [30], [33], [34]. It may also shed some light on the debate itself. Naturally, we are aware of the limitations of our study, in particular the fact that only one type of model has been analyzed here. Nevertheless we would like to emphasize that when building ABMs we should take into consideration which of the factors – personal traits or situation – determines the behavior in a particular situation.

## Supporting Information

Dataset S1 **Monte Carlo Simulation results and Matlab codes for** **Figure 2** **.**
(ZIP)
