# Supplementary figures and images for: Is the Person-Situation Debate Important for Agent-Based Modeling and Vice-Versa?
Source: PLoS One. 2014 Nov 4;9(11):e112203. doi: 10.1371/journal.pone.0112203 (PMC4219838; doi:10.1371/journal.pone.0112203)

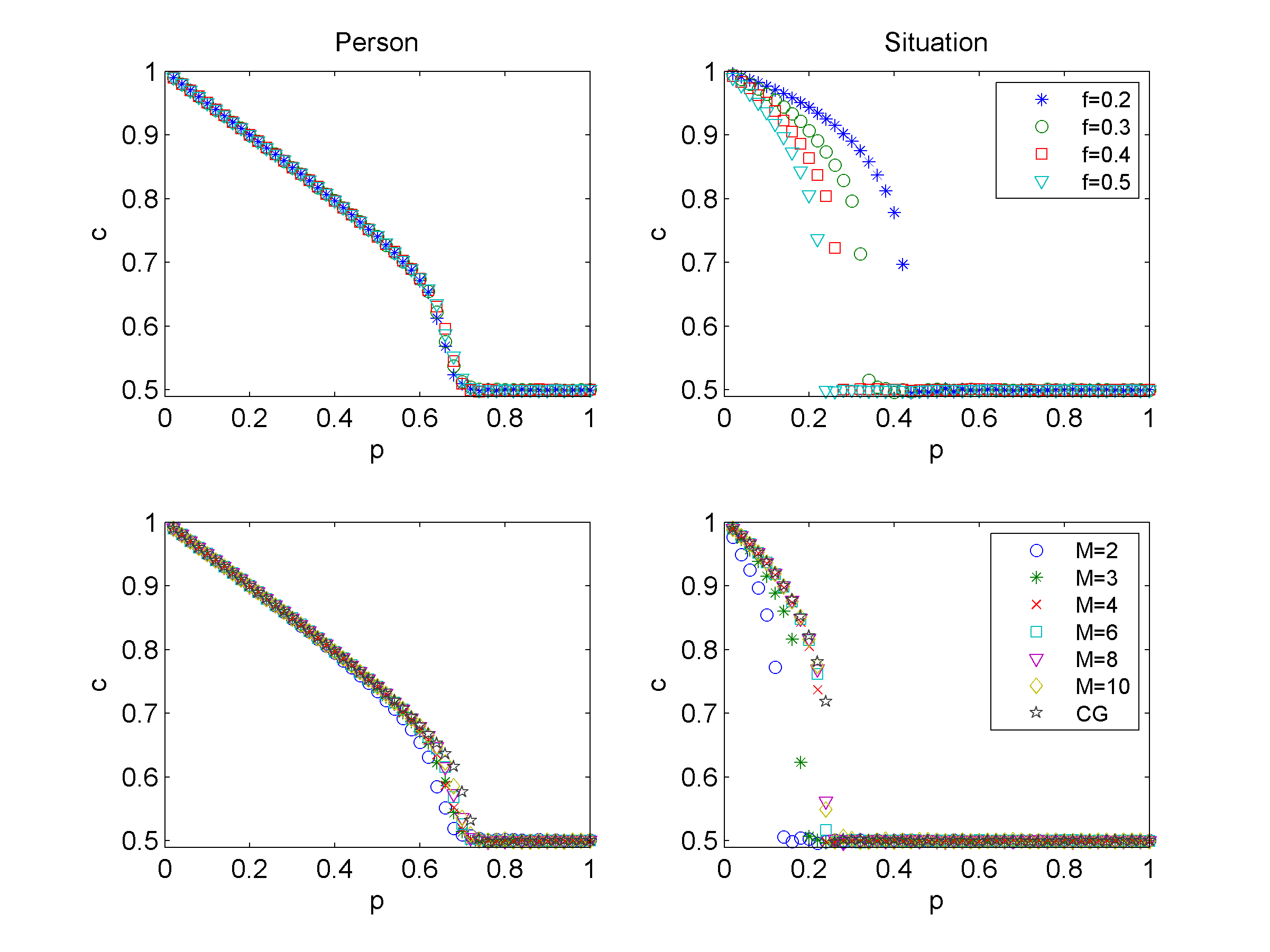

Supplement: Dataset S1 — Monte Carlo Simulation results and Matlab codes for Figure 2 . (ZIP) [file pone.0112203.s001.zip › fig2.png]
